# Takotsubo Syndrome Triggered by Acute Myocardial Infarction

**DOI:** 10.7759/cureus.70417

**Published:** 2024-09-29

**Authors:** Pablo González Alirangues, Verónica Artiaga de la Barrera, Carlos García Jiménez, Carolina Ortiz Cortés

**Affiliations:** 1 Cardiology, Hospital Fundación Alcorcón, Alcorcón, ESP

**Keywords:** apical akinesia, myocardial infarction, stemi, stress cardiomyopathy, takotsubo syndrome

## Abstract

Takotsubo syndrome (TTS) is a reversible heart failure syndrome that frequently manifests with symptoms and electrocardiogram (ECG) changes that mimic those of an acute myocardial infarction (AMI). Historically, coronary artery disease (CAD) has been regarded as an exclusion criterion in the diagnosis of TTS. However, recent reports have challenged this viewpoint, suggesting that the two conditions may coexist. The present case series provides evidence that not only is TTS able to coexist with CAD, but that an acute coronary syndrome (ACS) can act as a trigger for the development of TTS.

## Introduction

Takotsubo Syndrome (TTS), also known as apical ballooning syndrome, 'broken heart syndrome,' or stress cardiomyopathy, represents a significant form of acute reversible myocardial injury. It is characterized by transient regional systolic left ventricular dysfunction (more often involving apical segments) that can be associated with potentially fatal conditions such as malignant arrhythmias and cardiogenic shock. It is most frequently observed in postmenopausal women following exposure to emotional or physical stressors. Clinically, TTS commonly presents with symptoms and signs that mimic those of acute myocardial infarction (AMI), including the sudden onset of chest discomfort, dyspnea, electrocardiogram (ECG) changes such as ST-segment or T-wave alterations, and mildly elevated cardiac enzyme levels. It was previously considered to be a relatively benign condition due to its reversible nature. However, current evidence suggests that the mortality rate in-hospital is comparable to that associated with acute coronary syndrome (ACS) [1]. This underscores the significance of accurate diagnosis and appropriate management strategies.

The precise pathophysiology of TTS is not yet fully understood but may be associated with catecholamine elevations during periods of emotional or physical stress, and it is thought to have minimal overlap with that of the more prevalent atherothrombotic ACSs. Diagnostic criteria for TTS commonly employed in clinical practice include the absence of coronary artery disease (CAD) [2,3]. Nevertheless, in contrast to these criteria, we present two cases in which ST-elevation ACS served as the precipitating factor for TTS.

## Case presentation

Case 1

**Figure 1:**
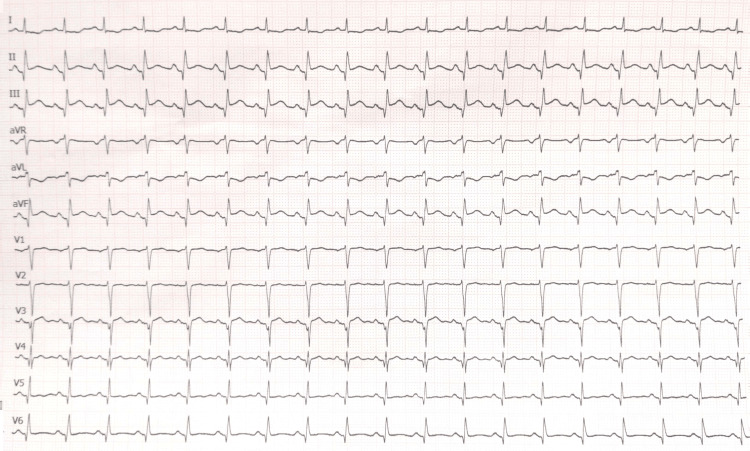
Initial ECG Both Q wave and ST elevation are seen in the inferior leads.

**Figure 2:**
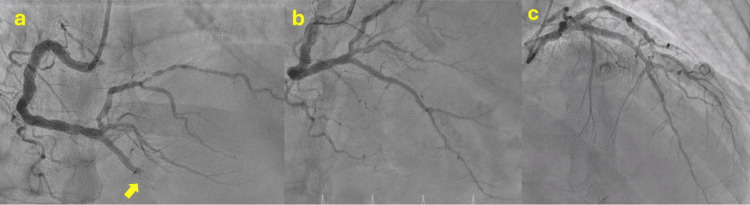
Coronary angiography a: Thrombotic occlusion of the PDA (yellow arrow); b: The result after drug-eluting stent implantation; c: The left coronary artery displays some atheromatosis but no significant obstructive lesions PDA: Posterior descending artery

**Figure 3:**
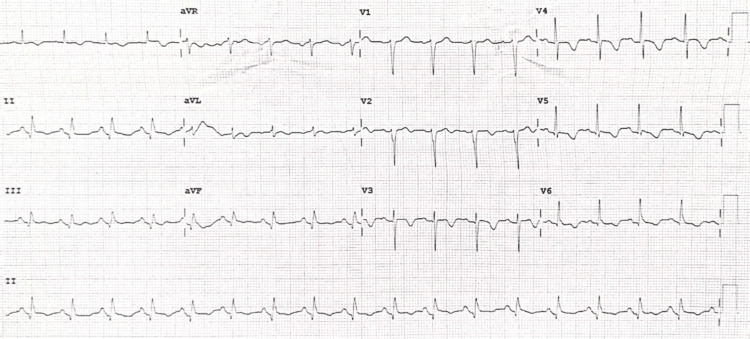
ECG performed after PCI Seen are Q waves in the inferior leads and shallow negative T waves. PCI: Percutaneous coronary intervention

**Figure 4:**
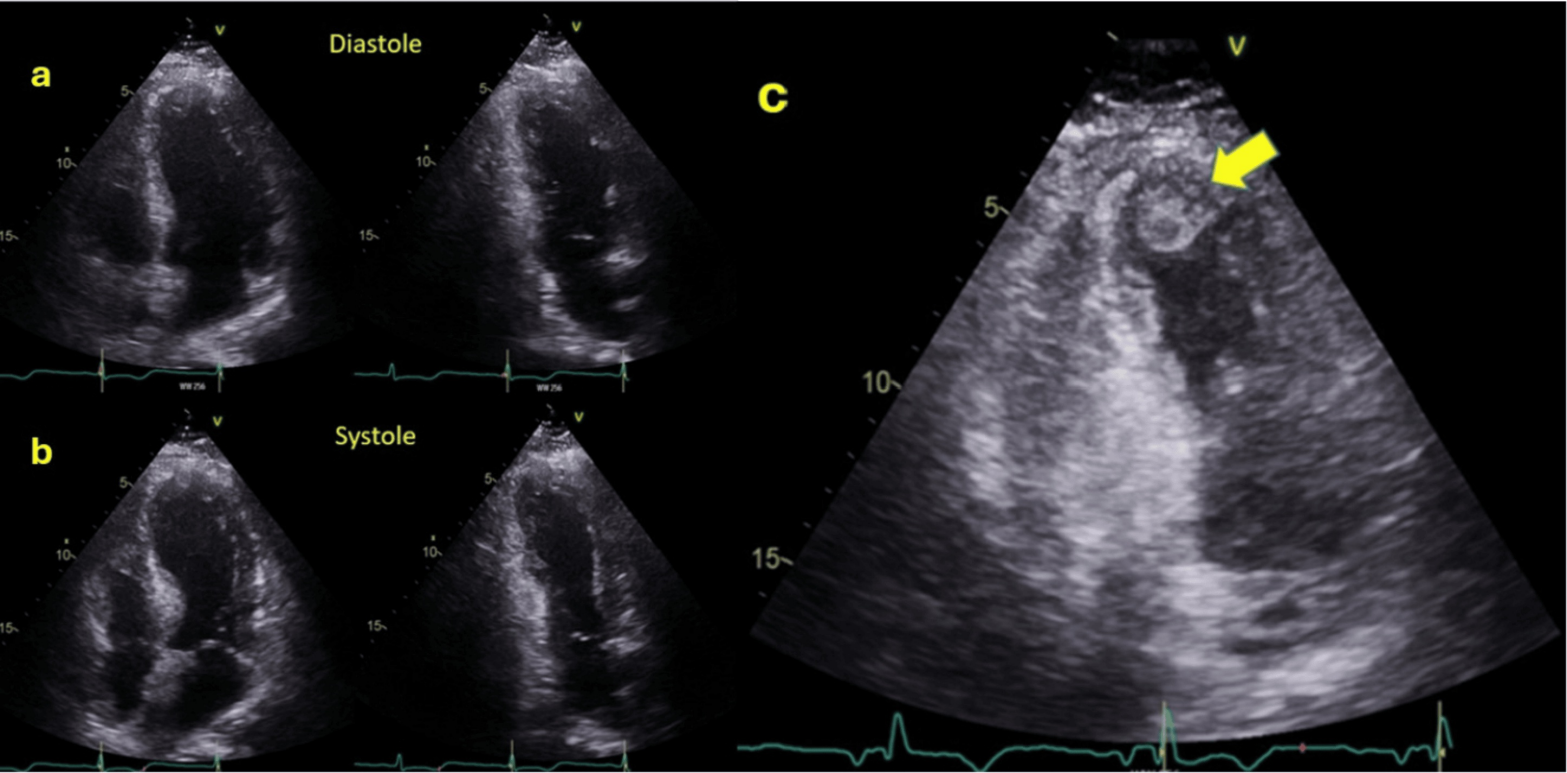
Echocardiogram performed 24 hours after PCI showing moderate left ventricular systolic dysfunction with akinesia of the mid-apical segments and LV apical trombus (yellow arrow) a: LV diastole; b: LV systole, revealing akinesia of the mid-apical segments; c: LV apical thrombus (yellow arrow) PCI: Percutaneous coronary intervention, LV: Left ventricle

A 56-year-old male with a history of hypertension, dyslipidemia, smoking, and aneurysmal subarachnoid hemorrhage secondary to an anterior temporal artery aneurysm was transferred to our hospital with a chief complaint of mid-chest pain and general discomfort that had persisted for five hours. An urgent ECG performed at the hospital where the patient first presented, showed ST elevation in the inferior leads (Figure 1). Upon arrival, the echocardiogram revealed akinesia in all mid-apical segments. An urgent coronary angiography was performed and revealed right dominance and thrombotic occlusion of the posterior descending artery (PDA), which was treated with drug-eluting stent implantation (Figure 2). The ECG performed after percutaneous coronary intervention (PCI) showed Q waves in inferior leads and shallow negative T waves (Figure 3). The patient remained clinically stable throughout his hospitalization and did not experience any recurrence of chest pain. Myocardial damage markers reached a peak at 45,000 ng/L of ultrasensitive troponin I (Tn I) and 1600 U/L of creatine phosphokinase (CPK). A repeat echocardiogram confirmed the presence of moderate left ventricular systolic dysfunction, with akinesia of the mid-apical segments on all sides and an image highly suggestive of a small apical thrombus in the left ventricle (LV). This prompted the initiation of anticoagulant therapy (Figure 4).

**Figure 5:**
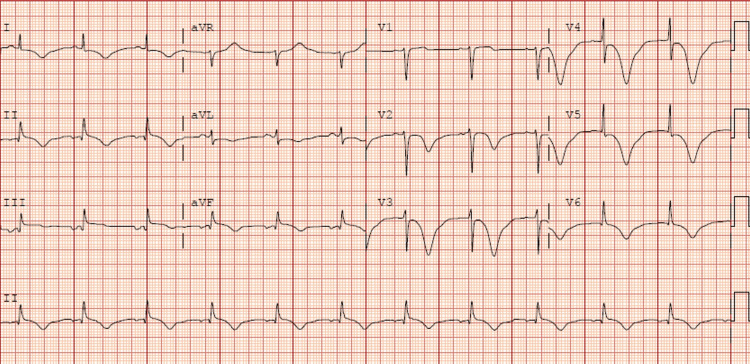
Evolution of ECG towards T wave negativization and marked QTc prolongation

**Figure 6:**
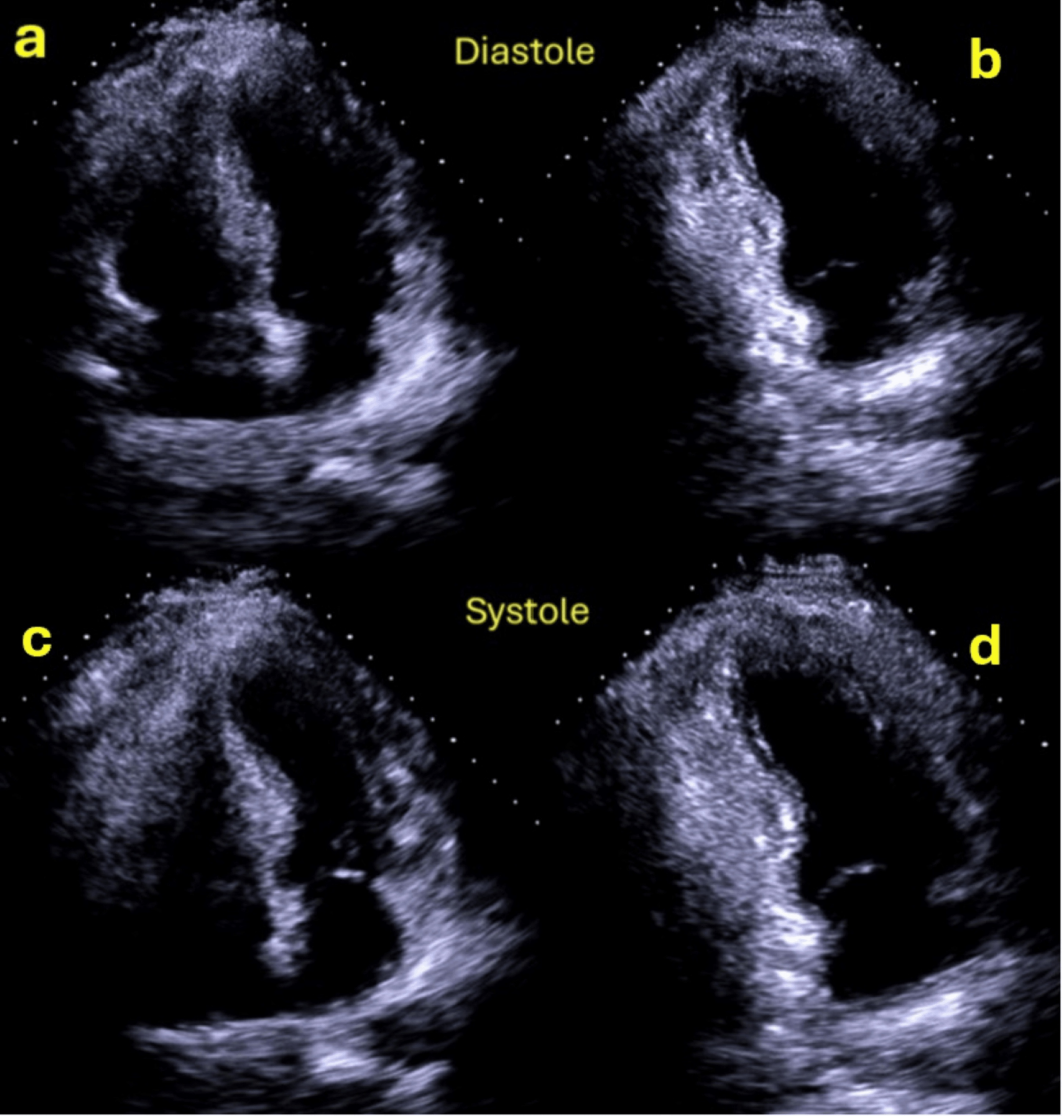
Echocardiogram performed at discharge showing improvement in segmental contractility alterations a: LV diastole apical four-chamber view; b: LV diastole apical two-chamber view; c: LV systole apical four-chamber view revealing improvement in segmental contractility alterations; d: LV systole apical two-chamber view also showing improvement in segmental contractility alterations LV: Left ventricle

In the ECG evolution, the patient exhibited the development of small Q waves in the inferior leads and a generalized inversion of the T wave, which was deep and symmetrical and associated with marked QTc interval prolongation (Figure 5). A subsequent echocardiogram conducted prior to discharge demonstrated a notable improvement in segmental contractility alterations, except for strict apical akinesia. In light of these findings (ECG changes and reversible extensive alterations in segmental contractility that were not contingent on the territory irrigated by the PDA), the diagnosis was rendered as TTS, potentially precipitated by AMI, as the patient refuted recent physical or emotional stressful events. Subsequent outpatient follow-ups demonstrated the normalization of ECG and echocardiographic abnormalities (Figure 6).

Case 2

**Figure 7:**
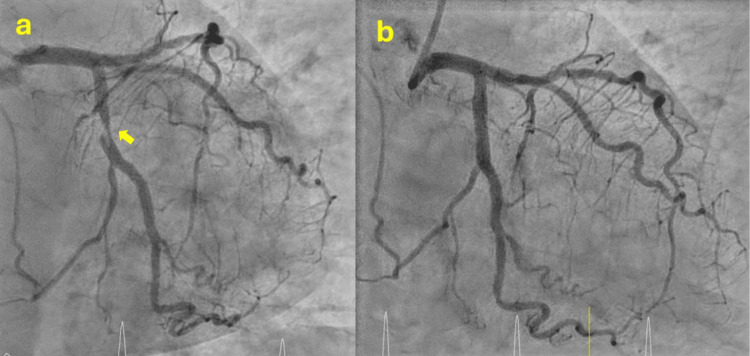
Coronary angiography a: Severe lesion in the proximal circumflex with complicated plaque image (yellow arrow); b: Final result after drug-eluting stent implantation

**Figure 8:**
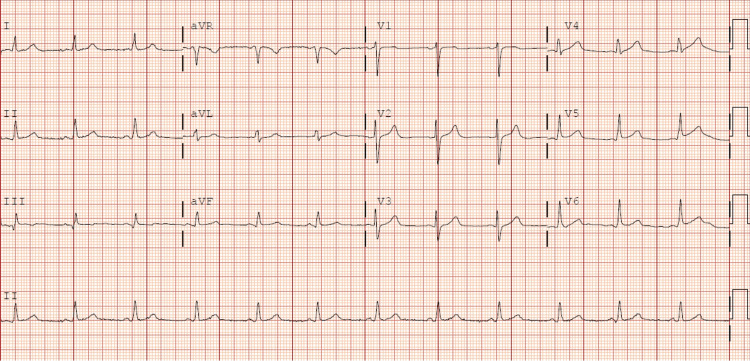
The ECG after PCI shows submillimetric ST elevation in inferior and lateral leads PCI: Percutaneous coronary intervention

**Figure 9:**
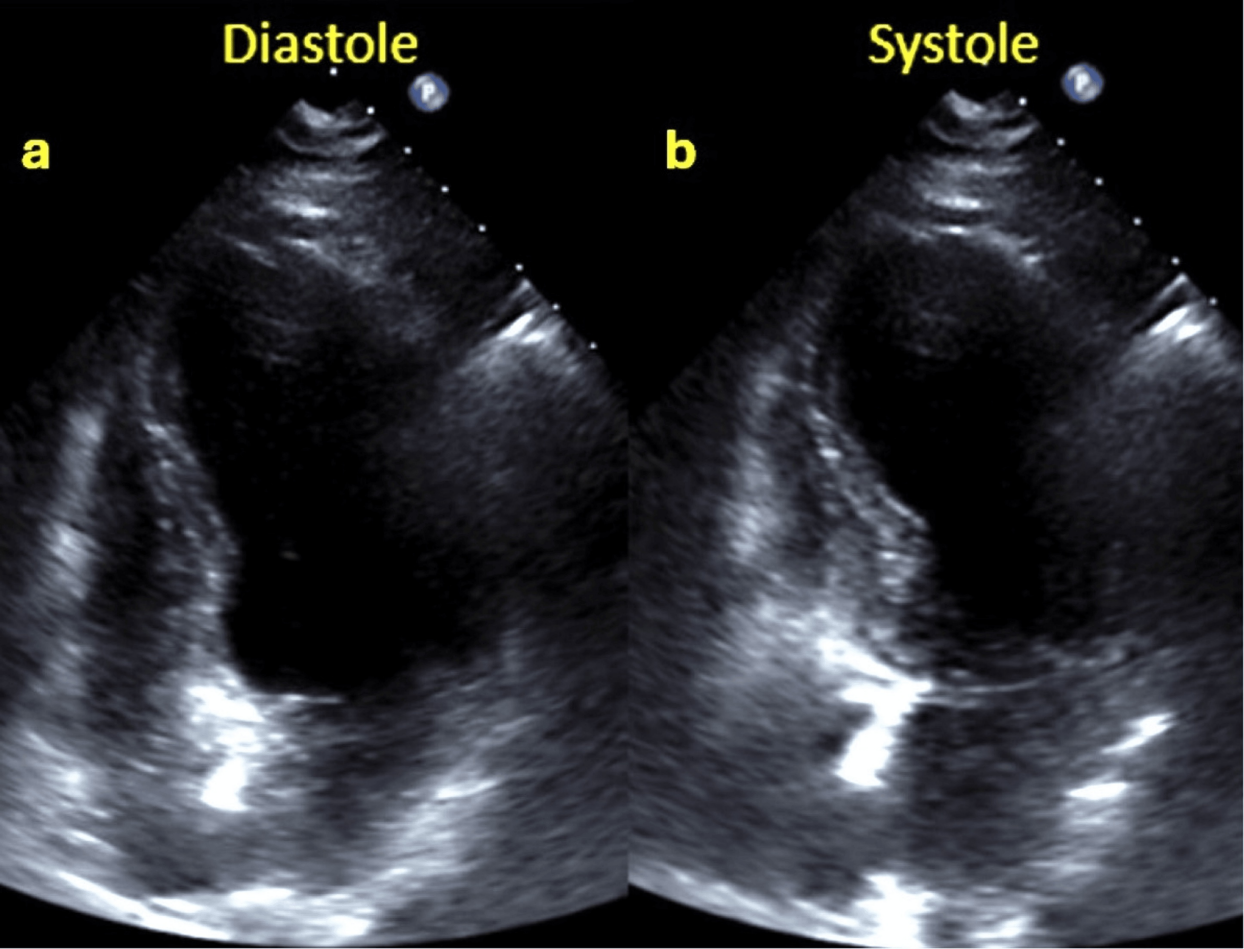
Echocardiogram after PCI shows moderate left ventricular systolic dysfunction and akinesia of the middle and apical segments of all sides a: LV diastole apical four-chamber view; b: LV systole apical four-chamber view showing akinesia of the middle and apical segments of all sides PCI: Percutaneous coronary intervention, LV: Left ventricle

A 77-year-old woman with a history of smoking, hypertension, diabetes, and dyslipidemia was transferred to the hospital with a complaint of three hours of chest pain. The ECG revealed ST elevation in the inferior and lateral leads. Emergent coronary angiography revealed right dominance and a severe lesion with a thrombus in the proximal circumflex. A drug-eluting stent was implanted, with successful results (Figure 7). No additional noteworthy lesions were observed in the coronary arteries. Persistent submillimetric ST elevation in inferior and lateral leads was evident in the post-coronary ECG (Figure 8). The urgent echocardiogram demonstrated moderate left ventricular systolic dysfunction, with an estimated ejection fraction of 35% and akinesia of the middle and apical segments of all sides. No other notable findings were observed (Figure 9). The subsequent evolution was favorable, with no new episodes of chest pain. Peak levels of myocardial damage markers were observed at an early stage, with CPK reaching 418 U/L and ultrasensitive Tn I 12,450 ng/L.

**Figure 10:**
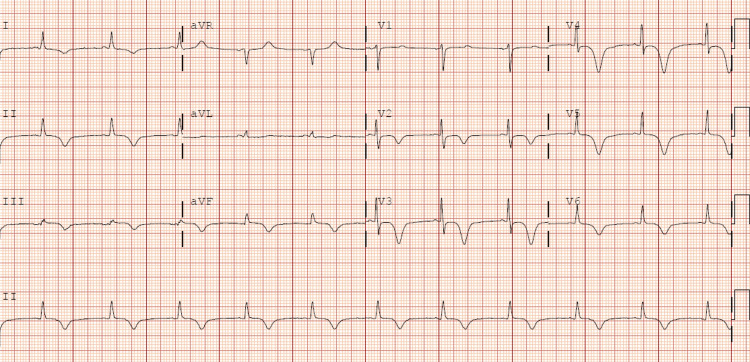
Evolution of the ECG during hospital admission towards T wave negativization and QTc prolongation

**Figure 11:**
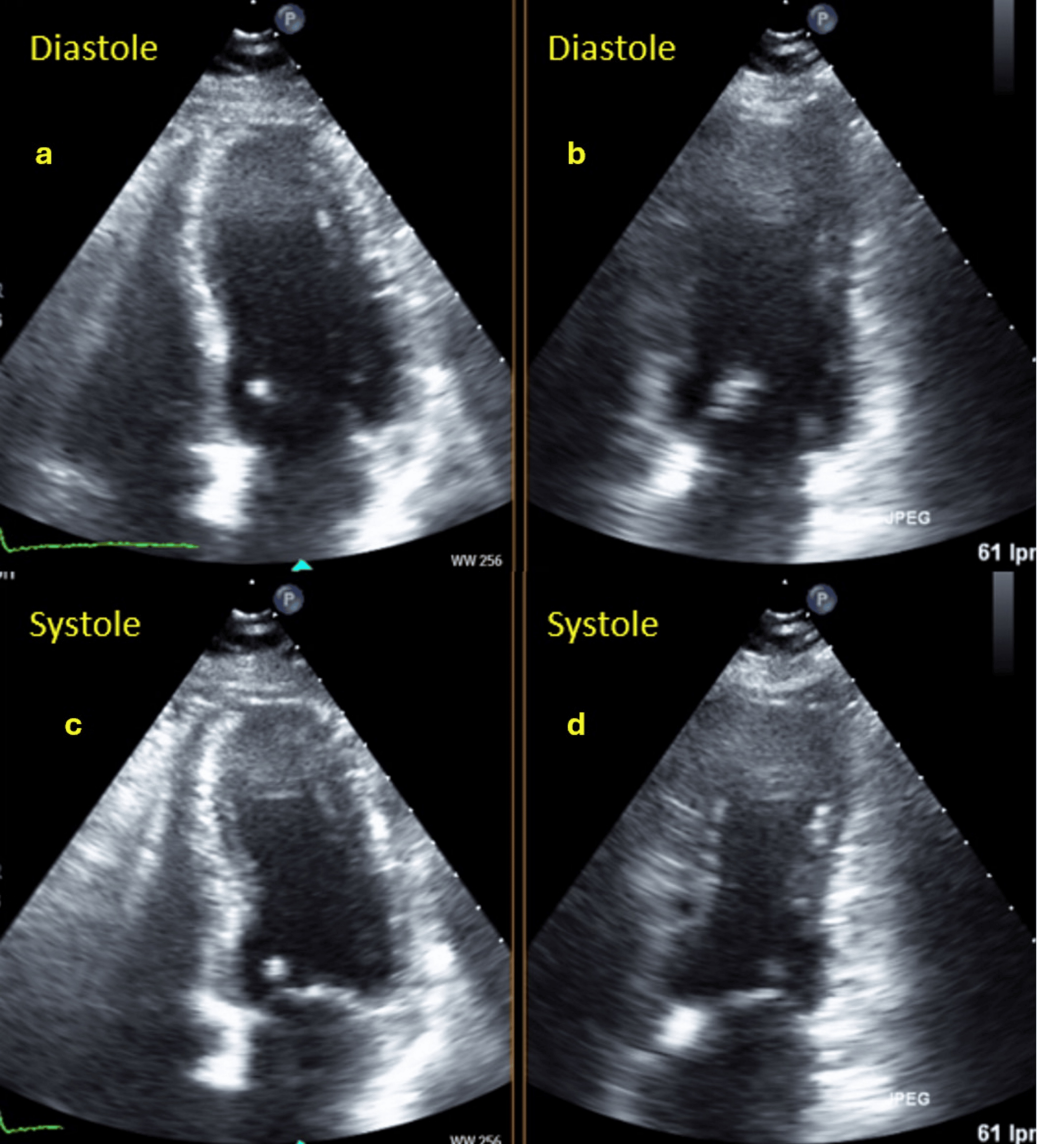
Echocardiogram at discharge shows correction of the alterations in the contractility of the middle segments while maintaining apical akinesia a: LV diastole apical four-chamber view; b: LV diastole apical two-chamber view; c: LV systole apical four-chamber view; d: LV systole apical two-chamber view LV: Left ventricle

**Figure 12:**
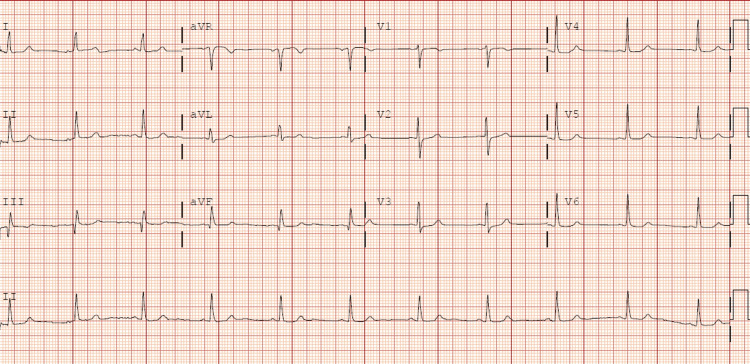
Follow-up ECG after discharge showing T wave and QTc normalization with small Q wave in III and augmented vector foot (aVF)

The ECG manifested a progression towards generalized T wave negativization and QTc prolongation (Figure 10). A repeat transthoracic echocardiogram was performed within the subsequent 48 hours, which demonstrated normalization of left ventricular systolic function and correction of the alterations in the contractility of the middle segments while maintaining apical akinesia (Figure 11). These findings were suggestive of TTS, which was probably induced by the inferolateral AMI, as the patient did not report any other physical or emotional stressors. Follow-up outpatient care showed normalization of electrocardiographic (Figure 12) and echocardiographic abnormalities.

## Discussion

Several scales have been developed for the diagnosis of TTS, including those proposed by the European Heart Failure Society (EHS) and the Mayo Clinic. Both scales require the absence of significant CAD for TTS diagnosis. However, a high percentage of patients presenting with TTS undergo coronary angiography, which frequently reveals the coexistence of coronary lesions. This is attributable to the epidemiological characteristics of the disorder, which predominantly affect older women [4,5] (additionally, diabetes mellitus has been identified as a risk factor for TTS [6]). In such instances, the previously outlined criteria may create uncertainty and potentially preclude the diagnosis in patients who actually present with TTS. We consider the international takotsubo (InterTAK) diagnostic criteria [7] to offer a more accurate assessment in this scenario, since they do not rule out the possibility of a TTS diagnosis in the presence of significant CAD.

Recent epidemiological literature on TTS indicates that the prevalence of coronary heart disease in patients with TTS ranges from 10% to 60% [4,8-10]. The Nationwide Inpatient Sample (NIS-USA) database, which analyzed almost 25,000 patients, revealed that almost 45% of patients with TTS had concomitant CAD [11]. It is therefore of great importance to investigate the relationship between the two entities since, as reported, it is not uncommon to find patients with TTS presenting concurrently with CAD. In such cases, it is assumed that three principal clinical scenarios may occur [12].

The first and most probable scenario is that CAD is a coincidental finding without a causal relationship with the onset of TTS, acting as a bystander. As previously reported, CAD is relatively common in patients with TTS; however, the evidence supporting a causal relationship between myocardial ischemia and the development of TTS remains limited. A review of the literature indicates that in approximately 80% of patients with TTS, a stressor trigger can be identified, the majority of which is physical rather than emotional [4,13]. Among the most common physical stressors are central nervous system disorders, surgical procedures, and infections. It is uncommon for TTS to be secondary to a cardiac condition, and even more so to an acute coronary event [13].

A second potential scenario in patients with TTS and concomitant CAD, though unlikely, is that of TTS precipitating an ACS [14]. There is a paucity of evidence to support this possibility, which we consider highly unlikely. Finally, a third potential scenario may exist in which an AMI serves as a precipitating factor for TTS development, functioning as a physical stressor. This has been described in some case series, and we believe our work supports this possibility [15-17]. Notably, in most cases documented in the literature, the artery responsible for the infarction was the left anterior descending artery (LAD). There are remarkably few cases where TTS was secondary to a coronary lesion in a different location, as illustrated by the two cases reported here.

In both patients presented in our study, the ECG changes and extensive segmental contractility abnormalities on the echocardiogram cannot be attributed exclusively to CAD. Conversely, the amount of stunned myocardium in both patients was found to extend beyond the supply region of the affected coronary artery. The progression of both the ECG and echocardiogram provides further evidence supporting the diagnosis of TTS. This is evidenced by the presence of generalized T wave negativization, QTc prolongation, and the presence of reversible left ventricular dysfunction caused by contractility alterations affecting all mid and distal segments, which is incongruent with an isolated inferior or inferolateral AMI. In both cases, no physical or emotional stressor was identified that could have acted as a trigger, making AMI the only evident cause for TTS. Regarding the elevation of ultrasensitive Tn I, it has limited utility in this scenario. In typical TTS patients, the elevation is modest; however, in AMI-induced cases, it is considerably higher due to myocardial damage secondary to the coronary event, as evidenced in our two cases.

This research aims to demonstrate that, despite CAD and TTS being traditionally regarded as distinct entities, there is mounting evidence to suggest that they may not be mutually exclusive. The coexistence of both entities is difficult to define and presents a significant diagnostic challenge that may potentially lead to uncertainty in treatment. Considering the two cases presented here, we propose that the presence of a potentially significant lesion on coronary angiography does not necessarily preclude the diagnosis of TTS. In such cases, the diagnosis should be guided by a comprehensive assessment of the patient's clinical presentation, with particular attention to ECG changes, the evolution of contractile abnormalities, and other findings from echocardiography and cardiac MRI. In the absence of any other identifiable trigger (emotional or physical), it is reasonable to hypothesize that an acute coronary artery lesion could be the underlying cause of TTS.

## Conclusions

The diagnosis of TTS is primarily one of exclusion, historically requiring the absence of CAD. However, recent registry data indicate a significant prevalence of CAD in patients with TTS. While CAD is often an incidental finding, an acute coronary event can precipitate TTS. This event more frequently involves the LAD but may also be associated with other coronary arteries. The presence of extensive reversible wall motion abnormalities beyond those expected from angiography, along with deep T wave inversion and marked QTc prolongation, suggests the superimposition of TTS on AMI. Further studies could confirm this, potentially leading to a modification of the diagnostic criteria for TTS.
